# The Role of Hypoxia-Inducible Factors (HIFs) in Triple-Negative Breast Cancer Radiation Resistance

**DOI:** 10.3390/ijms27219868

**Published:** 2026-11-04

**Authors:** Caden Caputo, Robert Richard Dingley, Yamamah Adel Al-Jumaili, Emma Mageau, Sujeenthar Tharmalingam, T.C. Tai

**Affiliations:** 1 Medical Sciences Division, Northern Ontario School of Medicine University, Sudbury, ON P3E 2C6, Canada; 2 School of Natural Sciences, Laurentian University, Sudbury, ON P3E 2C6, Canada; 3 Health Sciences North Research Institute, Sudbury, ON P3E 2H2, Canada

**Keywords:** triple-negative breast cancer, hypoxia, hypoxia-inducible factors, radiation, radiation resistance

## Abstract

Triple-negative breast cancer (TNBC) is an aggressive breast cancer subtype lacking estrogen receptor, progesterone receptor, and HER2 expression. Although ionizing radiation (IR) is widely used to treat TNBC, tumour radioresistance remains a major therapeutic barrier. Tumour hypoxia contributes to resistance by limiting IR-induced reactive oxygen species generation and activating pathways that promote tumour survival, progression, and aggressiveness. Hypoxia-inducible factors (HIFs) are oxygen-sensitive transcription factors that regulate many of these pathways; however, the mechanisms underlying HIF-mediated radioresistance in TNBC remain incompletely characterized. This review examines the role of HIF signalling in TNBC radiation resistance and emerging strategies for overcoming hypoxia-mediated radioresistance. PubMed and Web of Science were searched for studies investigating HIFs, TNBC, and radiation response. Relevant studies were screened against the eligibility criteria and analyzed for mechanistic findings and therapeutic interventions targeting HIF-associated pathways. The literature indicates that HIF activation promotes radioresistance through multiple mechanisms, including altered ferroptosis and pH regulation. Proposed countermeasures include pharmacologic inhibition of HIF signalling, nanotechnology-based delivery systems, and alternative radiation-based modalities, including photodynamic and radiodynamic therapies. Collectively, these findings identify HIF signalling as a central mediator of TNBC radioresistance and a promising therapeutic target for improving radiotherapy outcomes.

## 1. Introduction

Triple-negative breast cancer (TNBC) is a subtype of breast cancer that is characterized by the lack of estrogen receptors (ER) and progesterone receptors (PR), as well as the lack of human epidermal growth factor receptor 2 (HER2) [1]. TNBC is a highly aggressive and invasive subtype of breast cancer and poses significant therapeutic challenges given the absence of ER, PR, and HER2 as conventional therapeutic targets [2]. Accounting for 15–20% of all breast cancer diagnoses, this subtype has a poor prognosis in the metastatic setting, where median overall survival is reported to be 8–13 months [3]. TNBC is also associated with an increased risk of recurrence, typically due to resistance acquired throughout the treatment regimen [4]. Given this, the need for improved treatment strategies is crucial.

One of the current treatment options available for TNBC is radiation therapy. Specifically, ionizing radiation (IR) (X-rays and gamma rays) is used, as formed ions pass through the tissue and deposit energy into the cells, and consequently disrupt cell division by damaging genetic material [5]. Typically, radiation is delivered in fractionated doses with the intention of conserving the surrounding healthy tissue [5]. While radiation therapy holds great potential in treating cancer, repeated exposure may allow for a population of cells to survive and gain resistance, therefore compromising the success of the therapy [6]. Resistance to radiation in TNBC can result from several cellular and molecular processes, including cancer stem cells, epithelial–mesenchymal transition, DNA damage repair, redox regulation, dysregulated cell death and metabolic signalling [7,8]. These mechanisms are reviewed in Mageau et al., 2025 and Wang et al., 2025 [7,8].

One key driver of radiation resistance in TNBC is hypoxia [9]. Hypoxia in tumours develops as rapidly proliferating cells quickly outpace the development of the vasculature, resulting in inadequate oxygen delivery [10]. Tumour oxygenation is a key determinant of IR-induced cytotoxicity [11]. In radiotherapy research, this effect is known as the oxygen enhancement ratio (OER), in which oxygenated tumours are easier to treat with IR than hypoxic and anoxic tumours [11,12]. This arises from the physical constraints of IR, wherein oxygen is required to chemically fix radiation-induced DNA damage, and from hypoxia-mediated signalling that activates radioresistance pathways [12,13]. One benefit of radiotherapy in cancer treatment is the ability of IR to destroy tumour vasculature [14]. Given the tumour’s high dependence on its vasculature, vasculature destruction results in additional cell death [14,15]. Reactive oxygen species (ROS) and DNA damage generated as a result of IR initiate apoptotic and necrotic pathways in endothelial cells [14]. However, IR destruction of the vasculature also limits oxygen supply to the remaining tumour cells, selecting for pro-angiogenic cells and contributing to tumour recurrence [12,16]. Cellular response to tumour hypoxia is largely dependent on hypoxia-inducible factors (HIFs), a family of oxygen-sensitive transcription factors that function as master regulators of hypoxic adaptation [9]. HIFs are highly expressed in TNBC and are largely associated with the aggressiveness and therapy resistance associated with the disease [9,17]. HIF has also been shown to be upregulated following IR and regulates the adaptive radiation response through a number of mechanisms [15]. The goal of this review was to discuss recent advances in the study of HIF involvement in TNBC radiation resistance.

The HIF transcription factor family plays a vital role in the cellular response to low oxygen and is often termed the master transcriptional regulator of the cellular responses to hypoxia [18,19]. Given the prevalence of hypoxia, HIFs play a crucial role in the adaptive responses of tumour cells by transcriptionally activating over 100 downstream genes that regulate various pathways that are critical for cancer survival and progression [20]. HIFs are a class of constitutively expressed heterodimeric transcription factors that consist of HIF-α and HIF-β subunits, with HIF-α abundance regulated by oxygen-dependent degradation [18]. In humans, there are three paralogs of the HIF-α subunit: HIF-1α, HIF-2α (also known as endothelial PAS domain-containing protein 1 (EPAS1)), and HIF-3α [18,19]. There are two paralogs of the HIF-β subunit, Aryl Hydrocarbon Receptor Nuclear Translocator (ARNT) and ARNT2 [18]. The structure of HIFs is conserved, with several structural motifs shared by all members (Figure S1) [21].

HIFs are members of the basic helix–loop–helix Per-Arnt-Sim family of proteins (bHLH-PAS) [22]. Most bHLH-PAS proteins include a bHLH domain that facilitates DNA binding and two PAS domains, as well as a transrepression or transactivation domain [22]. In HIFs, the bHLH domain is responsible for DNA binding, whereas the PAS domains mediate the heterodimerization of HIF-α and HIF-β subunits [19,22,23].

HIF-1α and HIF-2α also contain an N-terminal transactivation domain (N-TAD) and a C-terminal transactivation domain (C-TAD) that are responsible for transcriptional activity [20]. The N-TADs of HIF-1α and HIF-2α determine gene target specificity [24]. Consequently, the differential activation of these HIF isoforms leads to the transcription of distinct yet overlapping sets of target genes. HIF-3α lacks the C-TAD, while retaining the N-TAD analogous to HIF-1α and HIF-2α [25,26]. HIF-1β and ARNT2 lack the N-TAD, but retain a C-TAD [27]. There are multiple important splice variants of HIF-3α with distinct regulatory functions [28].

**Figure 1:**
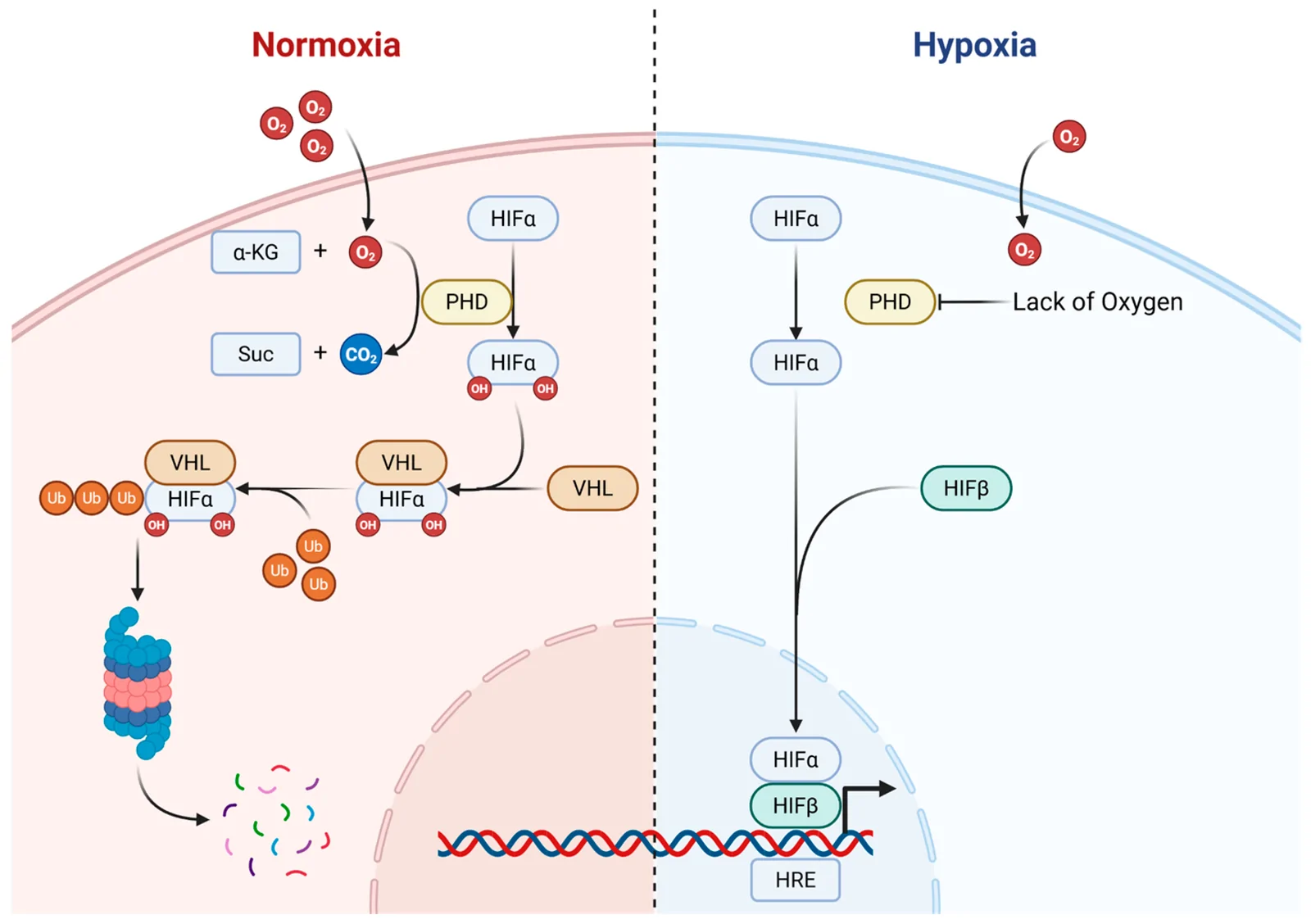
Schematic of the HIF oxygen-dependent degradation pathway. Under normoxic conditions, hypoxia-inducible factor alpha (HIF-α) subunits are rapidly degraded. In the presence of O_2_ and alpha-ketoglutarate (α-KG), the proline residues in the oxygen-dependent degradation domains (ODDD) of HIF-α subunits are hydroxylated by prolyl-4-hydroxylase domain (PHD) proteins, marking them for von Hippel–Lindau (VHL) E3 ubiquitin ligase-mediated destruction. Under hypoxic conditions, the low availability of O_2_ inhibits PHD activity, thereby causing the accumulation of HIF-α subunits, dimerization with HIF-1β subunits, nuclear localization, DNA binding, and transcriptional activation of target genes containing hypoxia response elements (HREs). Adapted from [31]. Created in BioRender. Dingley, R. (2026) https://BioRender.com/6t52y3t.

A key feature of the HIF-α subunits that differentiates them from HIF-β subunits is the oxygen-dependent degradation domains (ODDD) [29]. HIF-α subunits contain two ODDDs, the N-terminal and C-terminal ODDD (NODD and CODD) [30]. Each ODDD contains a highly conserved proline residue [29,30]. The ODDDs and specific proline residues are vital to the HIF regulatory response to oxygen content (Figure 1) [30].

HIF-α subunits are rapidly degraded under normoxic conditions, inhibiting HIF signalling [20]. HIF-1α has a half-life of about 5 min in normoxia [32]. Oxygen-dependent degradation of HIF-α is coordinated by interactions between the ODDD and prolyl hydroxylase domain (PHD) proteins [29,33]. There are four isoforms of PHDs (PHD1, PHD2, PHD3, PHD4), which catalyze the hydroxylation of the proline residues of the ODDDs of the HIF-α subunits in the presence of molecular oxygen, α-ketoglutarate, and iron (II) for catalytic activity [29,33,34]. The hydroxylation of these prolines promotes recruitment of the von Hippel–Lindau (VHL) protein and the E3 ubiquitin ligase, resulting in proteasomal degradation of the HIF-α subunit [20]. In addition to regulation at the ODDD, HIF-α subunits are further regulated by several other factors. Factor-Inhibiting Hypoxia-Inducible Factor (FIH) is a Fe^2+^/α-ketoglutarate dependent dioxygenase that acts as a negative regulator of HIF transcription (Figure S2) [35]. Under normoxic conditions, FIH catalyzes the hydroxylation of an asparagine residue in the C-TAD of HIF-α subunits, blocking cofactor recruitment and maximal transcriptional activation [35,36].

Together, these regulatory mechanisms allow HIFs to function as highly sensitive molecular oxygen sensors that coordinate the cellular response to hypoxia. Stabilization of HIF-α subunits under hypoxic conditions enables dimerization with HIF-β subunits, DNA binding at hypoxia response elements (HRE), and the transcriptional activation of a broad set of genes involved in metabolic adaptation, angiogenesis, proliferation, and survival [9,10]. Consequently, understanding the regulation and downstream effects of HIF signalling is essential for elucidating mechanisms of treatment resistance in hypoxic tumours.

The role of HIFs, particularly HIF-1α, in TNBC is well established in the literature, with HIFs regulating numerous cellular pathways that contribute to the aggressive phenotype of TNBC [9]. HIF-1α expression in TNBC is associated with poor prognosis and increased tumour progression [37,38]. Through the transcriptional activation of a broad network of downstream targets, HIF signalling promotes metabolic reprogramming, enhances DNA damage repair, stimulates angiogenesis, supports the enrichment of cancer stem cell populations, contributes to epithelial-mesenchymal transition (EMT), modulates apoptotic pathways, facilitates immune evasion, and drives extracellular matrix remodelling [38]. These broader pathways have been reviewed by Srivastava et al. and Liu et al. [10,38]. However, the involvement of these pathways in radiation resistance is not well known.

Hypoxia and HIF signalling have been well-established drivers of resistance to ionizing radiation [39]. TNBC tumours are frequently characterized by extensive hypoxia, high glycolytic metabolism, and elevated HIF signalling, all of which may contribute to treatment resistance and disease progression [40,41]. While numerous studies have investigated hypoxia-mediated radioresistance broadly, and others have explored the role of HIF signalling in breast cancer biology, research specifically addressing the intersection of HIF signalling, TNBC, and radiation response remains dispersed across the literature. To better define the molecular pathways and therapeutic strategies involved in HIF-mediated radiation resistance in TNBC, a systematic literature search was performed.

## 2. Literature Search and Study Selection

This review was informed by a structured literature search of PubMed and Web of Science on 27 February 2026. Utilizing the following search string: ((“Hypoxia-Inducible Factor*” OR “hypoxia inducible factor*” OR HIF OR HIF-1 OR HIF-2 OR HIF-3 OR HIF-1α OR HIF-2α OR HIF-3α OR EPAS1)) AND (“Triple Negative Breast Neoplasms” OR “triple negative breast cancer” OR TNBC OR “triple-negative breast cancer” OR (“breast cancer” AND (ER-negative OR PR-negative OR HER2-negative))) AND (“Radiotherapy” OR radiation OR radiotherapy OR radioresistance OR “radiation resistance” OR radiosensitivity OR “radiation response”)). In PubMed, the string was entered into the default search interface without field restrictions. In Web of Science, we ran a topic search using the same string. The searches returned 46 and 29 records, respectively.

Original studies were eligible if they: (1) investigated TNBC using cell, animal models, or clinical samples; (2) examined HIF expression, activity, regulation, or downstream signalling; (3) evaluated radiation response, an established radioresistant phenotype, or treatment strategies addressing hypoxia/HIF-associated radiation resistance. Studies involving multiple breast cancer subtypes were eligible when findings relevant to TNBC could be identified separately. During screening, we broadened the scope to include related treatment modalities, including radiodynamic therapy, photodynamic therapy, photothermal therapy, and modulated electrohyperthermia, in our review. We distinguished findings from these modalities from evidence directly demonstrating altered sensitivity to conventional radiotherapy. Direct experimental confirmation of HIF dependence was not required for inclusion; associations between HIF changes and treatment outcomes were distinguished from causally demonstrated mechanisms.

Studies were excluded if they did not provide identifiable TNBC-specific findings, did not examine HIF or did not address tumour biology in the context of radio- or other similar therapy. We excluded reviews, editorials, commentaries, and retracted publications from the included-study synthesis. Other relevant publications were consulted separately to provide background and support interpretation.

**Figure 2:**
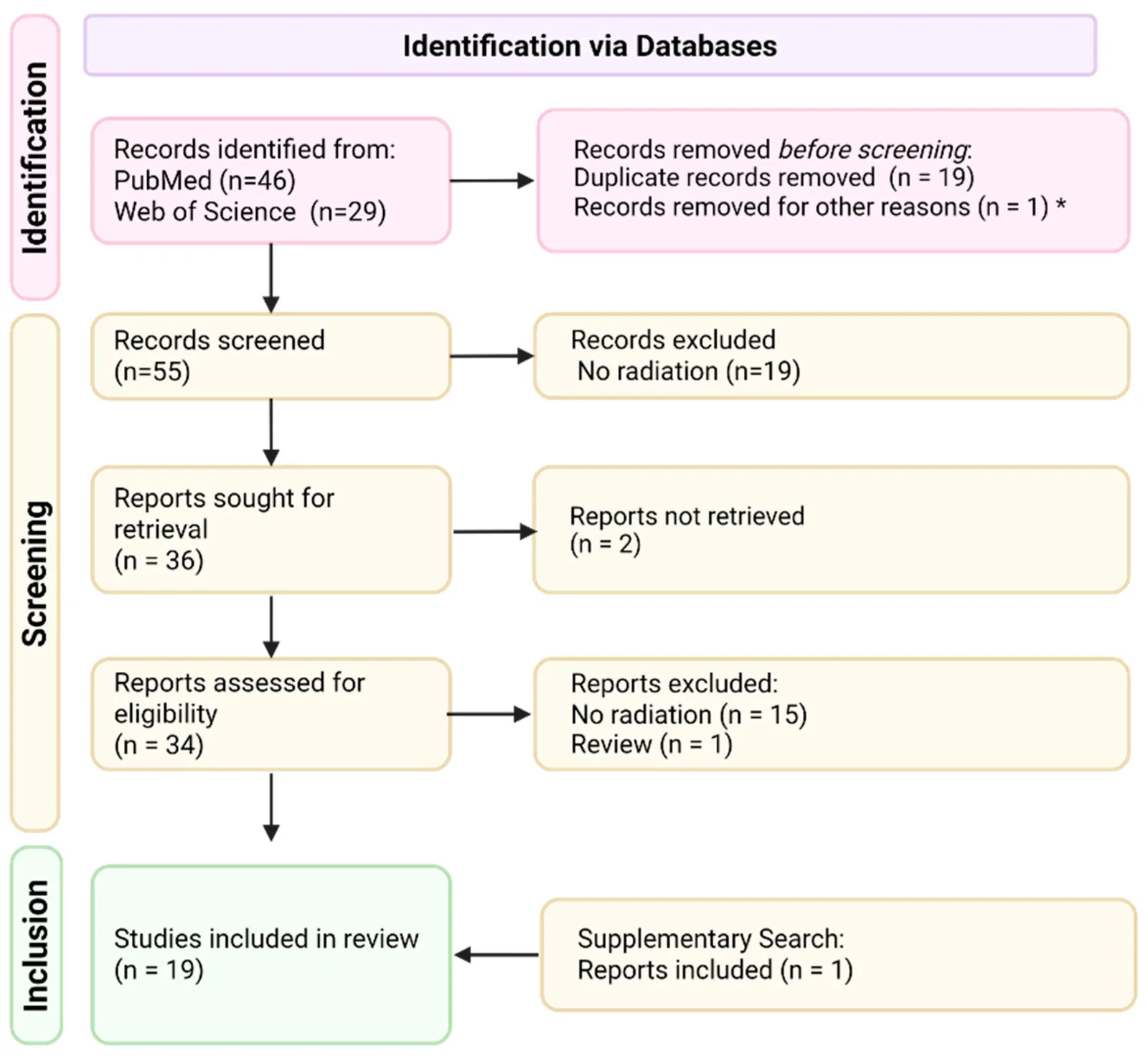
Adapted PRISMA 2020 flow diagram for new systematic reviews. The diagram summarizes record identification, screening, retrieval, and eligibility assessment, including supplementary searching during revision. * Retracted Articles. Created in BioRender. Dingley, R. (2026) https://BioRender.com/o3jn365.

Search results were combined, and duplicate records were removed using Zotero. Titles and abstracts were screened by C.C. and R.R.D. against the eligibility criteria. C.C. and R.R.D. conducted full-text assessments of potentially relevant reports and recorded reasons for exclusion. Disagreement was resolved through discussion and consultation with T.C.T. and S.T. Publications consulted solely for background context were distinguished from studies included in the evidence synthesis. During manuscript revision, targeted informal supplementary web searches were conducted to investigate reviewer-raised questions concerning HIF signalling, PDCD4, and FAK in TNBC radiation response. Additional records were assessed against the original eligibility criteria, resulting in the inclusion of one additional study (Figure 2).

## 3. Results and Discussion

The 19 included studies were heterogeneous in both their experimental models and research objectives. A full breakdown of each study included in this review can be found in Table 1. Some of the included studies examined molecular mechanisms by which HIF stabilization and downstream signalling contribute to radiation resistance, while others focused on therapeutic approaches intended, in part, to disrupt HIF activity and improve radiotherapy. The studies were largely preclinical, including TNBC cell lines and animal models. HIF-1α was the principal HIF isoform investigated, while comparatively few studies directly examined HIF-2α or HIF-3α. Although the review was initially focused on conventional ionizing radiation, a subset of the included studies examined related treatment modalities, including non-ionizing photodynamic therapy (PDT) and photothermal therapy (PTT), and X-ray-activated radiodynamic therapy (RDT). PDT relies on light-activated photosensitizers that transfer energy from non-ionizing radiation to molecular oxygen, generating ROS and inducing oxidative damage [42]. PTT utilizes photosensitizers that convert absorbed energy into localized heat, resulting in thermal ablation of tumour cells [43]. RDT uses X-rays to activate photosensitizers and generate ROS and exhibits better tissue penetration than PDT [44]. Some strategies also integrate multiple mechanisms to overcome hypoxia-driven resistance pathways. Targeted supplementary literature was also consulted to provide biological context, define emerging treatment modalities, and support interpretation of pathways that were not fully described within the 19 included studies. Despite their methodological diversity, the studies converged on four interconnected features of HIF-mediated radiation resistance in TNBC: (1) TNBC sustains HIF signalling through molecular chaperones and signalling loops; (2) HIF-driven metabolic reprogramming and the effects on radioresistance; (3) HIF-associated antioxidant responses and evasion of oxidative cell death; (4) the abnormal tumour vasculature and microenvironment as determinants of HIF-driven radioresistance.

### 3.1. Molecular Foundations of HIF Stability and Signalling

The fundamental prerequisite for HIF-mediated radioresistance is the sustained accumulation of HIF proteins, which are otherwise rapidly degraded via oxygen-dependent proteasomal pathways. Rather than functioning solely as a consequence of the hypoxic microenvironment, this stabilization in TNBC is actively reinforced by an integrated system of molecular chaperones and upstream signalling loops. Central to the cellular stress response is the molecular chaperone heat shock protein 90 (HSP90), which binds to and stabilizes a number of kinases and cellular factors, including HIF-1α [64,65,66]. While HSP90 is highly variable across breast cancers, elevated HSP90 expression is correlated with decreased survival rates in breast cancer patients [67]. HSP90 competes with receptor of activated protein kinase C (RACK1) for binding to the PAS-A domain of HIF-1α [68]. RACK1 binding to HIF-1α recruits the Elongin-C E3 ubiquitin ligase, resulting in PHD/VHL-independent proteasomal degradation of HIF-1α [68,69,70]. Therefore, HSP90 inhibits this oxygen-independent degradation of HIF-1α, which maintains the cell in a pseudohypoxic state [68]. Notably, HSP90 additionally binds to both HIF-2α and HIF-3α, with RACK1 overexpression also reducing HIF-2α protein levels [68,71]. Therapeutic strategies targeting this axis have demonstrated significant potential. Treatment of orthotopic TNBC mouse models with the HSP90 inhibitor Ganetespib has been shown to block HIF-1 activity, inhibiting tumour growth, vascularization, invasion, metastasis, and stem cell maintenance [72]. Targeting HSP90-HIF-1α in TNBC has also shown promise with non-ionizing combined photothermal and photodynamic therapy [43,58,73]. SNX2112 (HSP90 inhibitor) and IR825 (photosensitizer) were loaded into nanomicelles and combined for photothermal and photodynamic therapy [61]. The nanomicelles improved therapeutic efficacy by inhibiting HIF-1α accumulation via HSP90, resulting in increased ROS production [61]. Taken together, these studies suggest that HSP90 is a viable target to induce HIF-1α degradation and radiosensitization of TNBC. Interestingly, treatment of TNBC cells with KNK437, an inhibitor of stress-induced HSP synthesis, resulted in decreased HIF-1α accumulation and reversed hypoxia-induced radioresistance [74]. However, the observed mechanism involved inhibition of AKT and HIF-1α, rather than enhancement of HIF-1α degradation, suggesting a key role of other HSPs in HIF-1α signalling [74].

While chaperones like HSP90 provide immediate protection against HIF degradation, radioresistant TNBC cells utilize feed-forward signalling loops to amplify the hypoxic signal. A critical mediator in this context is endothelial cell-specific molecule 1 (ESM-1), also known as endocan, which is a secreted glycoprotein that has been implicated as an important effector molecule in angiogenesis and inflammation [75]. ESM-1 has been found to be significantly upregulated (~300-fold) in generated radiotherapy-resistant MDA-MB-231 (RT-R-MDA-MB-231) cells compared to parental cells [50]. RT-R-MDA-MB-231 cells exhibit increased proliferation, colony formation, migration, invasion, and adhesion; however, this aggressive phenotype was ameliorated following siRNA-mediated ESM-1 knockdown [50]. ESM-1 reinforces the resistant phenotype by activating the ERK1/2, PKC, and PDK1 pathways, which subsequently potentiate HIF-1α levels [50]. Our group similarly identified ESM-1 as a differentially regulated gene after transcriptomic analysis of RT-R-MDA-MB-231 cells [76]. Our results showed transcriptional changes associated with extracellular matrix remodelling, mitochondrial dysfunction, and epithelial–mesenchymal transition [76]. The involvement of ESM-1 is further validated in mouse xenograft models, where plasmid-mediated overexpression of ESM-1 drove increased tumour growth, metastasis, and angiogenesis [76]. High ESM-1 tumours exhibited increased HIF-1α expression, further confirming that ESM-1 acts as a critical mediator of TNBC radioresistance, in part by activating HIF-1α signalling [50,76].

While chaperones and upstream mediators ensure robust accumulation of HIFs, the long-term persistence of TNBCs’ radioresistance program is governed at the epigenetic level by epigenetic readers such as bromodomain-containing protein 4 (BRD4) [52]. BRD4 is upregulated in tumour cells and has been linked to the promotion of various transcription factors, including hypoxia-inducible signalling, through interaction with the acetylated lysine residues of histone tails on chromatins [52]. In TNBC, BRD4 supports tumour cell survival pathways and proliferation, which contributes to an immunosuppressive tumour microenvironment and thereby indirectly promotes radiotherapy resistance [52]. In a syngeneic murine TNBC model, BRD4 inhibition combined with local radiotherapy produces significantly greater tumour growth delay, fewer metastases, and improved abscopal control when compared with either radiation therapy or BRD4 inhibitor alone [52]. At the molecular level, BRD4 inhibitor treatment led to significantly reduced HIF-1α expression within the tumour microenvironment, whereas radiotherapy alone did not significantly change HIF-1α levels [52]. The downregulation of HIF-1α through BRD4 inhibitors was further emphasized by decreased PD-L1 expression, reduced infiltration of CD68^+^/CD206^+^ M2 tumour-associated macrophages (TAMs), increased proportion of M1-like macrophages, and enhanced intratumoural CD8^+^ T-cells, all of which are reportedly regulated by HIF-1α [52]. These changes suggest that BRD4 inhibition leading to HIF-1α suppression mitigates radiation-induced immunosuppression and alters the tumour microenvironment to be more radiosensitive.

In summary, TNBC sustains HIF-mediated radioresistance through a robust regulatory network involving post-translational stabilization through HSP90, feed-forward signalling through ESM-1, and epigenetic maintenance through BRD4. Once stabilized, HIF complexes enact the broad transcriptional changes required for tumour survival and immune evasion.

### 3.2. HIF-Driven Metabolic Reprogramming

Metabolic adaptation represents a major component of HIF-mediated regulation in TNBC, shifting the tumour cells from oxidative phosphorylation to glycolysis, and broader metabolic rewiring that supports growth, invasion, and therapy resistance [77,78]. This switch is essential for survival in hypoxic environments, such as those in TNBC solid tumours [78]. Glycolysis supports continued ATP and NADPH generation despite its lower efficiency per glucose molecule when compared to oxidative phosphorylation, thereby sustaining cell growth and proliferation under oxygen limitation [79]. At the transcriptional level, HIFs reprogram metabolism by inducing a broad gene network that redirects carbon flux away from oxygen-dependent mitochondrial respiration and toward anaerobic glycolysis. Both HIF-1α and HIF-2α contribute to this process, although they exhibit distinct roles and timing in their metabolic responses [78,79]. This metabolic shift results in an increase in the production of lactate and other acidic metabolites [80]. Therefore, HIF-driven metabolic reprogramming must be accompanied by mechanisms that regulate intracellular pH to prevent metabolic acidosis.

One of the principal regulators of pH in hypoxic tumours is carbonic anhydrase IX (CA IX), a metalloenzyme responsible for the catalysis of carbon dioxide and water into bicarbonate and protons [81]. CA IX is a transmembrane enzyme overexpressed in many tumours that supports the maintenance of an intracellular alkaline pH in tumour cells and an extracellular acidic pH, resulting in tumour cell growth while inhibiting the growth of normal cells [82]. Maintenance of this pH imbalance also contributes to the upregulation of key survival pathways, conferring radioresistance to tumour cells [83]. CA IX has therefore been a target of therapy in many cancers [82,83]. CA IX is a transcriptional target of HIF-1α and is generally upregulated in response to hypoxia [82]. The expression of CA IX varies across breast cancer cell lines, with TNBC showing higher CA IX mRNA and protein levels [48]. Overexpression of HIF-1α and CA IX predicts poor outcome in early-stage TNBC [51]. Hypoxia strongly induced the expression of CA IX in all breast cancer cell lines [48]. Treatment of breast cancer cell lines with betulin sulfonamides designed to target and inhibit CAs induced cell death [48]. TNBC cells treated with betulin sulfonamides under hypoxic conditions showed increased radiosensitivity [48]. This result correlated with inhibited CA IX activity and protein levels, while HIF-1α protein levels remained unchanged [48]. This work highlights CA IX and hypoxic tumour pH as significant contributors to HIF-1α-mediated radioresistance, making CA IX a viable target for radiosensitization therapy.

In addition to pH regulation, HIF-driven glycolytic shift also supports radioresistance by influencing DNA damage repair in response to radiation-induced damage. Lactate was historically considered a metabolic waste product, but it is now recognized as a signalling metabolite capable of regulating proteins through a post-translational modification called lysine lactylation, garnering attention as a target in cancer therapeutics [84]. Lactylation of HIF-1α blocks VHL recognition, supporting stability, representing a positive feedback mechanism of HIF-driven metabolic reprogramming [85]. The addition of lactate to TNBC cells enhanced DNA repair following irradiation, while the inhibition of lactate production impaired DNA repair and increased apoptosis [54]. High lactate levels result in the lactylation of the DNA repair protein MRE11, a component of the MRE11-RAD50-NBS1 (MRN) complex that mediates homologous recombination repair [54]. Lactylated MRE11 enhances DNA repair efficiency, conferring radioresistance [54]. Histone Deacetylase 5 (HDAC5) acts as a delactylase that removes the lactyl group from MRE11, ameliorating the lactate-driven increase in DNA damage repair [54]. Saikosaponin D is the active component of Radix Bupleuri, a widely used medicinal herb mainly derived from *Bupleurum chinense* and *Bupleurum scorzonerifolium*, which has been shown to have a number of antitumour effects [86]. Treatment of TNBC with saikosaponin D results in an increase in both mRNA and protein expression of HIF-1α [54]. The increased HIF-1α expression was associated with increased HDAC5 expression, reducing lactate-driven radioresistance [54]. Interestingly, this study identifies a context in which HIF-1α activation may reduce radioresistance; however, it is unclear if HIF-1α is required for saikosaponin D-induced HDAC5 expression and radiosensitization. Future studies using HIF-1α knockdown or knockout, ideally with rescue experiments, are needed to confirm the role of HIF-1α in this pathway.

Ortho-topolin riboside (oTR) is a naturally occurring cytokinin riboside originally isolated from poplar leaves, whereas protocatechualdehyde (PA) is a phenolic aldehyde derived from medicinal plants such as *Salvia miltiorrhiza*, *Phellinus linteus*, and *Prunella vulgaris* [57]. In radio-resistant TNBC cells, co-treatment with low-dose oTR and PA produced a synergistic reduction in cell viability and significantly suppressed tumour growth in radio-resistant TNBC xenografts [57]. Multi-omics profiling identified HIF-1 signalling as the major pathway altered by oTR and PA treatment [57]. Within the HIF-1 axis, co-treatment decreased glucose and lactate levels and downregulated PRKCA and NECAB3, two positive regulators of glucose uptake and HIF-1α activity [57]. Co-treatment also reduced basal and compensatory glycolysis, ATP production, and mitochondrial respiratory reserve capacity [57]. These findings suggest that combined oTR and PA exert synergistic anti-proliferative effects in radio-resistant TNBC by suppressing HIF-1α-driven glycolytic metabolism signalling. However, because this study primarily examined established radioresistant models rather than the direct combination of oTR, PA, and ionizing radiation, further work is required to determine whether the compounds function as radiosensitizers during active radiotherapy.

Together, these studies highlight HIF-driven metabolic reprogramming as a core component of HIF-mediated radioresistance. TNBC cells utilize the shift to glycolysis not just as a mechanism to sustain cell growth and proliferation under hypoxia, but also to further HIF signalling and protect against radiotherapy. Disrupting these adaptations may also increase tumour-cell susceptibility to radiation-induced oxidative stress, providing a transition to the ROS-dependent death pathways discussed in the following section.

### 3.3. Evasion of Oxidative Death Through Apoptotic and Ferroptotic Resistance

Radiation therapy induces cell death through direct DNA damage and through the generation of ROS, initiating apoptotic pathways [87]. However, one of the common hallmarks of cancer is the inhibition of pro-apoptotic pathways, resisting programmed cell death, conferring radiation resistance [88]. Hypoxia and HIF signalling reinforce this phenotype in solid tumours, including TNBC, by reducing pro-apoptotic signalling while increasing anti-apoptotic signalling [89,90,91]. Through these effects, HIF-1α can raise the threshold of oxidative damage required to initiate cell death, thereby promoting tumour survival and treatment resistance [91]. Therapeutic strategies that either suppress this HIF-mediated adaptive response or generate oxidative damage that exceeds its protective capacity may therefore improve treatment response.

One strategy to overcome this resistance is to suppress the HIF-1α-mediated adaptive response. Chrysin (5,7-dihydroxyflavone) is a natural flavone with anti-inflammatory, antioxidant, and pro-apoptotic activities that has been widely investigated for its antitumour effects, including ROS-mediated apoptosis and inhibition of inflammatory signalling pathways [49,92]. It downregulates Cyclin D1 in TNBC models and induces apoptosis, in part through p53-dependent regulation of Bax and Bcl-2 [49]. Importantly, chrysin has also been shown to inhibit HIF-1α and VEGF expression in TNBC cells, making it an appealing natural agent to counter hypoxia-driven radioresistance [49,92]. A combination of chrysin and 4 Gy radiation showed reduced cell viability compared with radiation alone or chrysin alone [49]. Changes in survival signalling supported this radiosensitivity effect, with reduced STAT3 and Cyclin D1 signalling and increased pro-apoptotic responses following combination therapy [49]. Interestingly, 4 Gy radiation therapy increased the proportion of HIF-1α-positive cells, whereas the combination of chrysin and radiation therapy resulted in a significant decrease in HIF-1α expression compared to radiation alone [49]. Given that STAT3 activation can promote HIF-1α and Cyclin D1 expression, it is suggested that chrysin’s radiosensitization pathway involves the inhibition of STAT-3-dependent signalling [49,93]. These findings suggest that chrysin may counter both apoptotic resistance and the HIF-1α response induced by irradiation. However, it is noteworthy that clinical biopharmaceutical studies show chrysin absorption is poor due to low solubility and moderate permeability [94].

Green-synthesized silver nanoparticles (Ag-NPs) represent another strategy for restoring apoptotic signalling while inducing radiosensitization in TNBC [56]. In TNBC cells, spherical Ag-NPs combined with 8 Gy radiation produced a synergistic reduction in cell viability and an increase in apoptosis compared with either treatment alone [56]. Mechanistically, combination therapy upregulated p53 and Bax, downregulated Bcl-2, and increased the Bax/Bcl-2 ratio, consistent with mitochondrial apoptosis and p53-dependent radiosensitization [56]. Ag-NPs have been shown to suppress the function of HIF-1α and downstream genes [95]. Radiation therapy alone notably increased the number of HIF-1α-positive cells, indicating radiation-induced hypoxia signalling [56]. While Ag-NPs alone caused a non-significant increase in HIF-1α, the combination of Ag-NPs with radiation therapy reduced HIF-1α expression compared with RT alone [56]. These findings associate nanoparticle-mediated radiosensitization with reduced HIF-1α expression [56]. However, the precise molecular route to HIF-1α regulation remains unresolved and requires targeted mechanistic studies. It has therefore not been established whether suppression of HIF-1α is required for nanoparticle-mediated radiosensitization or occurs secondarily to increased oxidative stress and apoptosis. Future studies utilizing HIF-1α loss- or gain-of-function experiments could help distinguish between these possibilities. More direct suppression of HIF-1α has also been explored to increase oxidative cell death. While PDT has emerged as a therapeutic strategy for TNBC, the ROS-mediated stabilization of HIF-1α and subsequent signalling remain barriers to treatment efficacy [58]. This creates a biological challenge similar to that of ionizing radiation therapy, in which treatment-induced oxidative stress may simultaneously activate pathways that support tumour survival. To address this issue with PDT, a cationic porphyrin-grafted lipid microbubble loaded with HIF-1α small-interfering RNA (siRNA) has been developed [58]. The porphyrin group acts as a photosensitizer for PDT, while the HIF-1α siRNA reduces HIF-1α protein levels [58]. Porphyrins are arranged as conjugated ring systems that efficiently absorb light, enter a long-lived excited state, and transfer energy to oxygen, generating ROS [42]. The generated microbubbles are large enough to be visible with ultrasound, and ultrasound-targeted microbubbles result in nanoparticle formation, which improves drug delivery [58]. In TNBC cells, siRNA delivery resulted in ~80% knockdown of HIF-1α mRNA, and its combination with PDT increased cell death compared to PDT alone [58]. In mouse TNBC xenografts, combination PDT, siRNA, and ultrasound showed complete inhibition of tumour growth [58]. These findings highlight HIF-1α as a critical mediator of PDT resistance and demonstrate the value of combining oxidative damage with direct inhibition of the resulting adaptive response. Given IR’s reliance on ROS-mediated damage, similar delivery platforms could be developed to overcome HIF-dependent IR resistance. Future studies should explore the use of similar siRNA delivery systems on IR resistance.

In addition to apoptosis, ferroptosis is an important oxidative cell death pathway that may be exploited to overcome TNBC radioresistance. Ferroptosis is an iron-dependent, non-apoptotic form of regulated cell death triggered by excessive iron accumulation and increased lipid peroxidation, resulting in oxidative cell death [96]. Ferroptosis is tightly linked with hypoxia and HIF signalling [97]. HIF-1α is generally a negative regulator of ferroptosis in both normal and tumour cells, by reducing free iron, stabilizing antioxidant systems, and inhibiting ferritinophagy [97]. Conversely, HIF-2α acts as a positive regulator of ferroptosis, especially in tumour cells, where it increases free iron and supports lipid remodelling [97]. Inhibition of ferroptosis has been shown to provide protection against radiotherapy [98]. Therefore, targeting hypoxia and inhibiting HIF-1α to induce ferroptosis as a radiosensitization method has been studied [98].

Interestingly, TNBC is particularly sensitive to ferroptosis because of its distinct dependence on iron, lipid, and glutathione metabolism [99]. A ferroptosis potential index (FPI) was developed as a metric based on the expression of ferroptosis regulatory genes, classifying TNBC as the most susceptible subtype of breast cancer [62]. Therefore, the induction of ferroptosis is a promising strategy to overcome HIF-1α-induced radioresistance in TNBC. Treatment of TNBC cells with iron-saturated Lactoferrin (Holo-Lf) was shown to induce ferroptosis [60]. This response was less pronounced in an ER-positive breast cancer cell line [60]. This supports TNBCs’ intrinsic sensitivity to iron-dependent oxidative death. Holo-Lf downregulated HIF-1α expression and enhanced the radiosensitivity of tumours in TNBC mouse models [60]. Phototheranostic iron pyrite (FeS_2_) nanocrystals have also been developed to exploit the high FPI of TNBC [62]. These FeS_2_ nanocrystals initiated dual death pathways of apoptosis and ferroptosis following near-infrared irradiation (NIR) in TNBC cells [62]. Similar treatment suppresses primary and metastatic TNBC tumours in mouse models [62]. Interestingly, HIF-1α signalling pathways were activated post-treatment, likely as a result of increased ROS generated by FeS_2_ and NIR treatment [62]. Despite this activation, this combined apoptotic and ferroptotic response remained sufficient to inhibit tumour growth. This suggests that HIF-1α signalling was unable to impede the dual apoptotic and ferroptotic pathways, highlighting a promising approach to overcome HIF-1α signalling. However, because the FeS_2_ nanocrystals were activated by NIR, this dual approach needs to be validated separately against conventional radiotherapy.

More complex nanosystems have been designed that simultaneously generate ROS, decrease antioxidant signalling, and disrupt mitochondrial survival. Chemodynamic Therapy (CDT) utilizes nanomaterials to trigger Fenton or Fenton-like reactions within the acidic tumour microenvironment, converting H_2_O_2_ into hydroxyl radicals and O_2_ [100]. Unlike oxygen-dependent ROS-generating strategies, CDT reactions are effective in hypoxic tumour environments and can support RT and RDT [55]. A multifunctional, mitochondria-targeted nanosystem comprising several components has been designed to combine RT, RDT, and CDT to overcome TNBC hypoxia-mediated radioresistance [55]. The nanosystem includes AuCu alloy nanoparticles, which act as radiosensitizers, and Chlorin e6, which acts as a photosensitizer for radiodynamic therapy [55]. The Cu ions additionally act as a CDT agent, catalyzing Fenton reactions [55]. The Au atoms also bind to the thiol groups of glutathione, thereby inhibiting glutathione radical-scavenging activity [55]. Additionally, the nanosystem contains mercaptoethyl-triphenylphosphonium (TPP), which targets and disrupts the mitochondrial membrane, further inducing apoptosis [55]. In TNBC cells, the nanosystem generated significant ROS while reducing HIF-1α expression, promoting decreased viability under hypoxic conditions [55]. In TNBC mouse xenografts, the nanosystem and 4 Gy X-ray irradiation reduced tumour size to 2.6% of the control, with tumour growth almost completely suppressed [55]. By combining ROS generation, antioxidant depletion, and mitochondrial targeting, this platform addresses multiple mechanisms underlying TNBC anti-apoptotic signalling. However, the number of active components complicates the identification of molecular pathways that drive the decrease in HIF-1α expression and the resultant cell death.

Collectively, these studies demonstrate that HIF-mediated evasion of oxidative cell death is a core component of TNBC radiation resistance. HIF-1α enables TNBC cells to withstand ROS by suppressing apoptosis, limiting ferroptosis, and supporting antioxidant defences. Disruption of these adaptations, or the generation of ROS that exceeds the protective capacity programmed by HIFs, therefore enhances radiosensitivity, while improving tumour oxygenation may further weaken this resistance.

### 3.4. Vasculature Normalization and Oxygen Resupply

While preceding sections focused on the intrinsic mechanisms driving HIF-mediated radioresistance, this section focuses on the hypoxic tumour microenvironment. Upon activation in TNBC, HIF-1α and HIF-2α upregulate a broad angiogenic transcriptional program, targeting key effectors such as vascular endothelial growth factor A (VEGF-A), VEGF receptors (VEGFR-2 and VEGFR-3), angiopoietins, platelet-derived growth factor (PDGF), and fibroblast growth factors (FGFs) [38,78]. Under chronic hypoxia, HIF-2α maintains the expression of genes involved in long-term vascular remodelling (VEGF, PAI-1) [78]. Less studied is HIF-3α, though it acts mainly as a modulator for HIF-1/HIF-2 activity rather than a direct angiogenic driver [101]. While these pathways promote endothelial cell proliferation, migration, and vascular permeability to supply the growing tumour, the resulting vasculature in TNBC is often leaky and disorganized [78]. This dysfunctional angiogenesis worsens intratumoural hypoxia, impedes drug delivery, and facilitates invasion and metastasis [58,78]. Therefore, this section groups strategies that either repair this aberrant tumour vasculature to restore functional oxygen flow or that bypass the need for blood supply entirely through exogenous oxygenation, effectively dismantling the microenvironment that fosters radioresistance.

Conventional radiotherapy often induces acute vascular damage, exacerbating tissue hypoxia and triggering a robust HIF-mediated angiogenic rebound that ultimately supports tumour survival [46,101]. To counteract this, emerging strategies such as minibeam radiation therapy (MBRT) focus on vascular normalization [46,102]. MBRT is a spatially fractionated technique that delivers submillimeter parallel beams of radiation, preferentially damaging immature tumour vessels while sparing mature, healthy vasculature [46,102]. In TNBC models, MBRT reduces radiation-induced hypoxia, downregulates HIF-1α-driven angiogenic pathways, and induces transient vascular permeability, leading to enhanced tumour control and drug delivery compared to conventional radiotherapy [46,102]. This highlights MBRT as a promising developing technique to treat TNBC while avoiding damage to healthy vascularization and hypoxia-induced resistance that develops following conventional radiotherapy. Radiation-associated changes in the surrounding tissue may also influence HIF signalling and tumour progression. Higher-dose IR of TNBC mouse xenografts was associated with increased decorin expression in peritumoral muscle, reduced tumour growth and metastasis, increased programmed cell death protein 4 (PDCD4) expression, and decreased HIF-1α expression [59]. These findings suggest that stromal responses may contribute to the effects of radiotherapy; however, the study did not establish whether HIF-1α suppression was required for these effects.

Similarly, pharmacological interventions can prevent the angiogenic recovery that typically follows radiotherapy-induced vascular stress. Modulated electrohyperthermia (mEHT) creates targeted tumour damage through thermal and electromagnetic effects but can inadvertently trigger HIF-1α-mediated revascularization [45]. However, the concurrent administration of the cardiac glycoside, digoxin, effectively blocks this compensatory mechanism [45]. Digoxin has shown potential to be a repositioned HIF-1α inhibitor [45]. By preventing the accumulation of HIF-1α, digoxin halts the downstream signalling cascade required for endothelial recruitment [45]. This suppression translates directly into a functional inhibition of tumour revascularization, as evidenced by significantly reduced expression of CD31, a critical marker of endothelial density [45]. These findings support a model in which mEHT induces acute vascular stress while digoxin prevents the HIF-1α-mediated adaptive response that promotes tumour survival. By suppressing HIF-1α and limiting HIFs’ abnormal angiogenic recovery, digoxin may help maintain tumours in a metabolically stressed or more radiosensitive state.

For tumour regions where natural vasculature is too compromised to be normalized, active oxygenation strategies which bypass the biological blood supply altogether can be utilized to generate oxygen in situ. Similar to the oxygen-dependent mechanisms of radiotherapy, photodynamic therapy (PDT) relies heavily on local molecular oxygen to generate cytotoxic ROS [63]. Consequently, applying PDT to an already hypoxic TNBC tumour exacerbates the hostile microenvironment, initiating a vicious cycle of oxygen consumption that further stabilizes HIF-1α [63]. To break this cycle, one approach uses an injectable oxygen-generating phototherapy hydrogel (POP-Gel) containing Chlorin e6, CaO_2_, and catalase [63]. Upon injection, CaO_2_ reacts with H_2_O to produce H_2_O_2_, which catalase subsequently converts into O_2_ [63]. In TNBC xenografts, this sustained localized oxygen delivery significantly decreased hypoxic regions for up to five days, minimized HIF-1α expression, and enhanced PDT efficacy, ultimately suppressing tumour growth and metastasis [63]. The POP-Gel and PDT also stimulated an immune response, as evidenced by increased IFN-γ levels and CD8^+^ T-cell infiltration [63]. Integrating similar sustained oxygen-delivery methods presents a highly promising avenue to bypass vascular limitations and prevent hypoxia-induced radioresistance.

Ultimately, the structural normalization of the tumour microenvironment and the mitigation of hypoxia serve to overcome direct radioresistance and to reverse immune evasion. An immunosuppressive tumour microenvironment can limit antitumour immune responses in TNBC [103]. This evasion is heavily driven by HIF-1α and its associated overexpression of the LDHA isoenzyme, LDH5, as a result of HIF-driven metabolic reprogramming [47]. In TNBC, HIF-1α and LDH5 expression correlates with fewer tumour-infiltrating lymphocytes and an overall poor clinical prognosis [47]. To bridge the gap between vascular repair and immune activation, a biomimetic photothermal therapy (PTT) is being utilized to actively remodel the hypoxic niche [53]. PTT can induce immunogenic cell death (ICD), triggering the release of damage-associated molecular patterns that stimulate immune infiltration [104]. Building on this, a targeted biomimetic nanoparticle was recently designed to combine PTT with direct vessel normalization in TNBC [53]. The system uses a 4T1 tumour cell membrane coating for targeting, a polydopamine core for near-infrared (NIR) heat generation, and gambogic acid (an HSP90 inhibitor), thereby inhibiting hypoxia-mediated angiogenesis [53]. Under NIR, the nanoparticle generated significant heat in vitro and showed significant cell death; classical ICD marker release was observed [53]. In 4T1 mouse xenograft models, the nanoparticle and PTT significantly reduced tumour growth, showed prolonged survival, reduced lung metastases, induced vessel normalization, and improved the immune microenvironment [53]. This was driven in part by increases in tumour oxygenation and a reduction in HIF-1α expression, resulting in decreased hypoxic angiogenesis [53]. The combination of nanoparticle and PTT with immunotherapy further increased tumour inhibition, decreased metastasis, and improved survival [53]. This work highlights that PTT, combined with vascular normalization, is an effective method to overcome poor immune infiltration in hypoxic TNBCs.

Taken together, these studies establish that an aberrant, hypoxic vascular niche is a core driver of TNBC radiation resistance. The HIF-mediated angiogenic response enables TNBC cells to survive radiotherapy by maintaining a disorganized, oxygen-depleted, and immunosuppressive microenvironment. Disruption of this through structural normalization or the localized generation of oxygen enhances radiosensitivity while subsequently facilitating immune cell infiltration to further improve tumour sensitivity.

## 4. Conclusions

**Figure 3:**
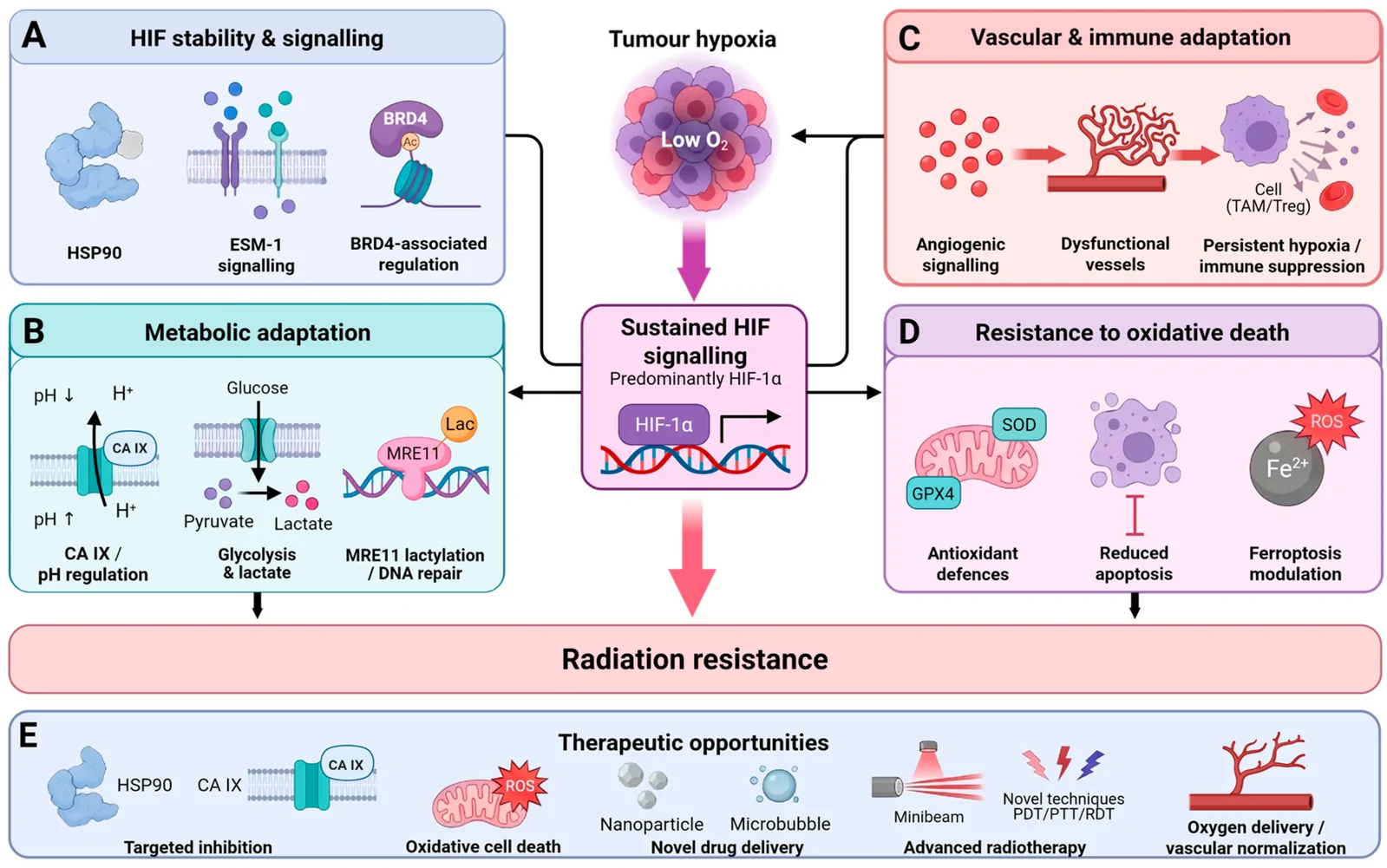
Mechanisms of HIF-mediated radiation resistance and emerging therapeutic strategies in TNBC. The hypoxic tumour microenvironment promotes sustained HIF signalling, predominantly through HIF-1α, driving radiation resistance across multiple cellular domains. (**A**) HIF stability and signalling are maintained by chaperones (HSP90), feed-forward signalling loops (ESM-1), and epigenetic maintenance (BRD4). (**B**) Metabolic reprogramming shifts cellular metabolism towards glycolysis, requiring pH regulation via CA IX and enhancing DNA damage repair through lactate-driven MRE11 lactylation. (**C**) Vascular and immune adaptation produces a dysfunctional vascular network that maintains the hypoxic and immunosuppressive environment. (**D**) Tumour cells evade radiation-induced oxidative death via increased antioxidant defences, reduced ferroptosis, and apoptosis suppression. (**E**) Therapeutic opportunities to overcome hypoxia-driven resistance include targeted inhibition of HIF mediators (HSP90 and CA IX), novel nanoparticle and microbubble delivery systems, minibeam radiotherapy and related photodynamic, photothermal, and radiodynamic approaches, and strategies focused on direct oxygen delivery and vascular normalization. Created in BioRender. Caputo, C. (2026) https://BioRender.com/uyi2ndx.

This review provides a comprehensive analysis regarding the role of HIFs in radiation resistance in TNBC (Figure 3). The available evidence implicates several interconnected mechanisms, including HIF stabilization through HSP90 and ESM-1 signalling, metabolic reprogramming, altered ferroptotic and apoptotic responses, and maintenance of the hypoxic tumour microenvironment. Accordingly, HIF-associated pathways represent promising targets for radiosensitization. The reviewed studies also highlight emerging strategies for overcoming hypoxia- and HIF-mediated resistance, including direct and indirect inhibition of HIF-1α, local oxygen generation, nanomaterial-based drug delivery, minibeam radiation therapy, and related ROS- or heat-based approaches, such as photodynamic, photothermal, and radiodynamic therapy.

Despite these promising findings, key limitations exist within the literature. Most therapeutic approaches remain in the early preclinical stage. Many of the nanoparticle and multimodal treatment studies used small in vivo cohorts, which is common in exploratory research but limits confidence in treatment efficacy, toxicity, and reproducibility. Larger studies, independent validation, and more clinically representative models are therefore required before these approaches can be translated into treatment. Greater distinction is also needed between interventions that directly enhance conventional radiotherapy and those studied only with alternative modalities such as PDT or PTT. Differences in experimental models in the reviewed studies limit comparison across studies. The included studies did not assess differential HIF expression and downstream signalling across the various models (in vitro and in vivo) used under matched conditions; therefore, their equivalence cannot be assumed. Findings should be interpreted in the context of the model and exposure conditions used.

Importantly, the literature remains heavily focused on HIF-1α, while the contributions of HIF-2α and HIF-3α to TNBC radiation response remain poorly defined. None of the studies included in this systematic review directly examined the role of HIF-3α in TNBC radiation resistance. However, this gap reflects a broader limitation in hypoxia research, as the regulatory functions of HIF-3α and its numerous isoforms have only comparatively recently begun to be rigorously investigated. Given the distinct and potentially context-dependent functions of each HIF family member, future studies should incorporate isoform-specific loss-of-function, gain-of-function, and rescue experiments to distinguish their contributions to radioresistance.

Several important mechanisms remain underexplored in TNBC, despite supporting evidence of HIF signalling’s role in radiation resistance. In particular, few studies have investigated the role of HIF-mediated DNA damage repair, EMT, and cancer stem cell (CSC) enrichment with respect to radiation resistance. Hypoxia-driven alteration in DNA damage repair, mediated in part by HIF signalling, may contribute to the high level of genomic instability that is characteristic of TNBC. CSCs are a subpopulation of tumour cells which have the capacity for self-renewal, tumour initiation, and differentiation. For these reasons, CSCs are suspected to drive tumour recurrence, metastasis, and therapy resistance. With these processes being highly relevant to treatment response, future research should focus on their interactions with HIF signalling and radiation resistance. Additionally, future research should also address tumour heterogeneity, acute versus chronic hypoxia, interactions between HIF isoforms, and the influence of the immune and vascular microenvironments. Overall, the evidence identifies HIF signalling as an important mechanistic link between tumour hypoxia and radiation resistance in TNBC. Continued investigation of this axis may support the development of more effective, personalized strategies to improve radiotherapy response.

To conclude, growing evidence suggests that HIFs play a large role in mediating radiation resistance in TNBC through the regulation of multiple cellular processes. Overall, this study highlighted HIF signalling as a key mechanistic link between tumour hypoxia and radiation resistance in TNBC, as well as emerging therapeutic strategies aimed at overcoming radiation resistance. Continued investigation of this axis may guide the development of more effective and more personalized therapeutic strategies.

## Figures and Tables

**Table 1:** Characteristics and main findings of individual studies included in the systematic review.

Article Title	Reference	Main Findings	Model System	Radiation/Energy-Delivery Parameters
Digoxin-Mediated Inhibition of Potential Hypoxia-Related Angiogenic Repair in Modulated Electro-Hyperthermia (mEHT)-Treated Murine Triple-Negative Breast Cancer Model	Bokhari et al. 2024 [45]	Repeated mEHT induced hypoxia-driven angiogenic responses in TNBC, while combination with digoxin reduced hypoxia, angiogenesis, and tumour growth, enhancing mEHT efficacy.	4T07, 4T1 cells, and female BALB/c mice	13.56 MHz of mEHT.
Minibeam radiation therapy remodels tumour microenvironment and suppresses HIF-1α/VEGFR axis to overcome radioresistance in triple-negative breast cancer	Fang et al. 2025 [46]	MBRT reduced tumour hypoxia and remodelled the tumour vasculature, accompanied by suppression of HIF-1α/VEGFR signalling and improved tumour control.	Female BALB/c mice	MBRT (150 Gy) or conventional radiotherapy (CRT, 7 or 15 Gy).
Hypoxia and anaerobic metabolism relate with immunologically cold breast cancer and poor prognosis	Giatromanolaki et al. 2022 [47]	High HIF-1α and LDH5 expression was associated with an immunologically cold tumour microenvironment and poorer prognosis.	Human breast cancer patients	3.5 Gy daily fractions x 10 days, with an additional boost of 2 × 4 Gy fractions.
Betulin Sulfonamides as Carbonic Anhydrase Inhibitors and Anticancer Agents in Breast Cancer Cells	Güttler et al. 2021 [48]	Betulin sulfonamides exhibited cytotoxic, antiproliferative, proapoptotic, and radiosensitizing effects in TNBC cells while regulating CA IX and CA XII expression.	TNBC cell lines: MDA-MB-231, Hs578T, and BT-20; comparator breast cancer cell lines: SK-BR-3 and T47D	Between 2 Gy and 14 Gy, depending on cell line and oxygen levels.
Synergistic Effect of Chrysin and Radiotherapy Against Triple-Negative Breast Cancer (TNBC) Cell Lines	Jafari et al. 2023 [49]	Chrysin enhanced the response of MDA-MB-231 cells to radiation, accompanied by increased apoptosis and reduced radiation-associated HIF-1α expression.	Human TNBC MDA-MB-231 cells	Total dose of 4 Gy photon 6 MV with a dose rate of 300 cGy/min
ESM-1 Overexpression is Involved in Increased Tumourigenesis of Radiotherapy-Resistant Breast Cancer Cells	Jin et al. 2020 [50]	ESM-1 was overexpressed in radioresistant TNBC cells and activated HIF-1α, NF-κB, and STAT3 pathways to promote tumour progression.	Human TNBC MDA-MB-231 cells and radioresistant MDA-MB-231 cells	Fractionated irradiation: 25 fractions of 2 Gy (total dose 50 Gy) using a 6-MV photon beam.
Overexpression of HIF-1α and CAXI Predicts Poor Outcome in Early-Stage Triple Negative Breast Cancer	Jin et al. 2016 [51]	Combined HIF-1α and CAIX expression served as a prognostic marker for disease-free survival in early-stage TNBC, depending on treatment and basal phenotype.	Clinical TNBC patient data	N/A
BRD4 Inhibition Enhances the Antitumour Effects of Radiation Therapy in a Murine Breast Cancer Model	Kim et al. 2023 [52]	BRD4 inhibition enhanced radiotherapy efficacy, accompanied by reduced HIF-1α expression and changes consistent with a less immunosuppressive tumour microenvironment.	4T1-Luc murine TNBC cell line and female BALB/c mice	8 Gy per treatment, leading to irradiation with a total of 24 Gy.
Biomimetic Nanomodulators With Synergism of Photothermal Therapy and Vessel Normalization for Boosting Potent Anticancer Immunity	Lan et al. 2024 [53]	Treatment with a biomimetic nano system improved tumour hypoxia and vascular normalization, enhancing immune infiltration and photothermal therapy responses in TNBC.	4T1 cells and BALB/c mice	Irradiated with an 808 nm laser (1 W cm^−2^) for 6 min
Saikosaponin D Mitigates Radioresistance in Triple-Negative Breast Cancer by Inducing MRE11 De-Lactylation via HIF-1α/HDAC5 Pathway	Li et al. 2025 [54]	Saikosaponin D enhanced radiosensitivity and reduced MRE11 lactylation, accompanied by increased HIF-1α and HDAC5 expression.	Human TNBC cell lines MDA-MB-231 and MDA-MB-468	Dose rate of 200 MU/min, with doses ranging from 0 to 16 Gy.
Mitochondria-Targeted Nanosystem Enhances Radio–Radiodynamic–Chemodynamic Therapy on Triple Negative Breast Cancer	Li et al. 2023 [55]	Mitochondrial-targeted nanosystem enhanced radiotherapy in both normoxic and hypoxic TNBC by targeting mitochondria and increasing ROS-mediated tumour cell killing.	4T1 cells, BALB/c mice	0, 1, 2, 4, 8, and 16 Gy radiation doses.
Green-Synthesized Silver Nanoparticles From Peel Extract of Pumpkin as a Potent Radiosensitizer Against Triple-Negative Breast Cancer (TNBC)	Montazersaheb et al. 2024 [56]	Silver nanoparticles enhanced radiation-associated cell killing and apoptosis, accompanied by reduced HIF-1α expression compared with radiation alone.	Human TNBC MDA-MB-231 cells	4 and 8 Gy from 6 MV photons and a 300 cGy/min dose rate.
Multi-omics Profiling Reveals Ortho-topolin riboside and Protocatechualdehyde Combination Exhibits Anti-proliferative Activity by Modulating Metabolic Pathways in In Vitro and In Vivo Radio-resistant MDA-MB-231 cell models	Nam et al. 2025 [57]	Combined ortho-topolin riboside and protocatechualdehyde treatment synergistically reduced tumour growth and altered multiple metabolic pathways, including HIF-1 signalling, in radio-resistant TNBC.	Human TNBC MDA-MB-231 cells, BALB/c mice	2 Gy radiation for 25 cycles; experimental irradiation at 3 and 6 Gy.
Ultrasound-Targeted Photodynamic and Gene Dual Therapy for Effectively Inhibiting Triple Negative Breast Cancer by Cationic Porphyrin Lipid Microbubbles Loaded with HIF-1α-siRNA	Sun et al. 2018 [58]	Multifunctional cationic porphyrin lipid microbubbles loaded with HIF-1α siRNA enhanced TNBC therapy by suppressing HIF-1α expression, improving photodynamic therapy efficacy, and increasing tumour cell killing.	Human TNBC MDA-MB-231 cells, BALB/c nude mice	Irradiated with or without a PDT laser (λ = 650 ± 5 nm, 200 mW, 10 min)
Anti-Tumor Responses to Hypofractionated Radiation in Mice Grafted with Triple Negative Breast Cancer is Associated with Decorin Induction in Peritumoral Muscles	Yu et al. 2018 [59]	Higher-dose irradiation was associated with increased peritumoral muscle decorin, increased tumour PDCD4 expression, decreased HIF-1α expression, and reduced tumour growth and lung metastasis.	MDA-MB-231 cells and xenografts in female BALB/c nude mice	Single doses of 2 and 10 Gy (6 MeV electron beam, 3 Gy/min)
Holo-lactoferrin: The Link Between Ferroptosis and Radiotherapy in Triple-Negative Breast Cancer	Zhang et al. 2021 [60]	Holo-lactoferrin enhanced radiosensitivity in TNBC by relieving tumour hypoxia, increasing ROS generation, promoting ferroptosis, and enhancing radiation-induced DNA damage.	Human TNBC MDA-MB-231 and MCF-7 cells, Female BALB/c nude mice	4 Gy Radiation.
Suppression of the HSP90-HIF-1α Pathway with SNX2112-Encapsulated Nano-micelles for effective Triple-Negative Breast Cancer Photothermal Combined Photodynamic Therapy	Zhang et al. 2025 [61]	SIDR nano-micelles suppressed HSP90–HIF-1α signalling, enhanced photothermal and photodynamic therapy, and inhibited TNBC tumour growth.	Murine 4T1 breast cancer cells and Panc02 pancreatic cancer cells	808 nm laser (1 W/cm^2^) for 6 min each
Near-Infrared Phototheranostic Iron Pyrite Nanocrystals Simultaneously Induce Dual Cell Death Pathways via Enhanced Fenton Reactions in Triple-Negative Breast Cancer	Zhao et al. 2023 [62]	FeS_2_–PEG8 combined with NIR irradiation induced apoptosis and ferroptosis and inhibited tumour growth despite increased HIF-1α pathway activity.	Human breast cancer cell lines (MDA-MB-231, MDA-MB-453, BT474, MCF7) and the human pancreatic adenocarcinoma cell line (BxPC-3, control). and female BALB/c mice	808 nm NIR laser: 2.5 W/cm^2^ for 5 min (in vitro); 1.6 W/cm^2^ for 10 min (in vivo).
Inhibition of Breast Cancer Proliferation and Metastasis by Strengthening Host Immunity with a Prolonged Oxygen-generating Phototherapy Hydrogel	Zhou et al. 2019 [63]	POP-Gel improved tumour oxygenation, reduced HIF-1α and VEGF expression, and enhanced photodynamic immunotherapy to inhibit TNBC growth and metastasis.	4T1 cells, BALB/c mice	100 μL POP-Gel (1 mg/mL) + 660 nm laser at 100 mW/cm^2^ on days 1, 3, and 5

## Data Availability

No new data were created or analyzed in this study. Data sharing is not applicable to this article.

## Supplementary Materials

The following supporting information can be downloaded at: https://www.mdpi.com/article/10.3390/ijms27219868/s1.

## Abbreviations

The following abbreviations are used in this manuscript:

AKT	protein kinase B
ARNT	aryl hydrocarbon receptor nuclear translocator
bHLH	basic helix–loop–helix
BRD4	bromodomain-containing protein 4
CA	carbonic anhydrase
CAD	C-terminal transactivation domain
CDT	chemodynamic therapy
CODD	C-terminal oxygen-dependent degradation domain
CSC	cancer stem cell
C-TAD	C-terminal transactivation domain
EMT	epithelial–mesenchymal transition
EPAS1	endothelial PAS domain protein 1
ER	estrogen receptor
ERK	extracellular signal-regulated kinase
ESM-1	endothelial cell-specific molecule 1
FGF	fibroblast growth factor
FIH FPI	factor inhibiting HIF
	ferroptosis potential index
HDAC5	histone deacetylase 5
HER2	human epidermal growth factor receptor 2
HIF	hypoxia-inducible factor
Holo-Lf	holo-lactoferrin
HRE	hypoxia response element
HSP	heat shock protein
ICD	immunogenic cell death
IFN-γ	interferon gamma
IR	ionizing radiation
LDHA	lactate dehydrogenase A
LDH5	lactate dehydrogenase isoenzyme 5
lncRNA	long noncoding RNA
MBRT	minibeam radiation therapy
mEHT	modulated electrohyperthermia
MRN	MRE11-RAD50-NBS1 complex
NIR	near-infrared
NODD	N-terminal oxygen-dependent degradation domain
NP	nanoparticle
N-TAD	N-terminal transactivation domain
ODDD	oxygen-dependent degradation domain
OER	oxygen enhancement ratio
oTR	ortho-topolin riboside
PA	protocatechualdehyde
PAI-1	plasminogen activator inhibitor 1
PAS	Per-Arnt-Sim
PDT	photodynamic therapy
PDCD4	programmed cell death protein 4
PDGF	platelet-derived growth factor
PDK1	3-phosphoinositide-dependent protein kinase 1
PD-L1	programmed death-ligand 1
PHD	prolyl hydroxylase domain protein
PI3K	phosphoinositide 3-kinase
PKC	protein kinase C
PLK1	polo-like kinase 1
POP-Gel	prolonged oxygen-generating phototherapy hydrogel
PR	progesterone receptor
PRISMA	Preferred Reporting Items for Systematic Reviews and Meta-Analyses
PTT	photothermal therapy
RACK1	receptor for activated C kinase 1
RDT	radiodynamic therapy
ROS	reactive oxygen species
RT	radiation therapy
RT-R	radiotherapy-resistant
STAT3	signal transducer and activator of transcription 3
TAM	tumour-associated macrophage
TNBC	triple-negative breast cancer
VEGF	vascular endothelial growth factor
VEGFR	vascular endothelial growth factor receptor
VHL	von Hippel–Lindau
