# Supplementary material for: The Role of Hypoxia-Inducible Factors (HIFs) in Triple-Negative Breast Cancer Radiation Resistance
Source: Int J Mol Sci. 2026 Nov 4;27(21):9868. doi: 10.3390/ijms27219868 (PMC13764003; doi:10.3390/ijms27219868)
Supplement: Supplementary file 1 [file ijms-27-09868-s001.zip › ijms-4622073-supplementary.pdf]

Supplementary Materials

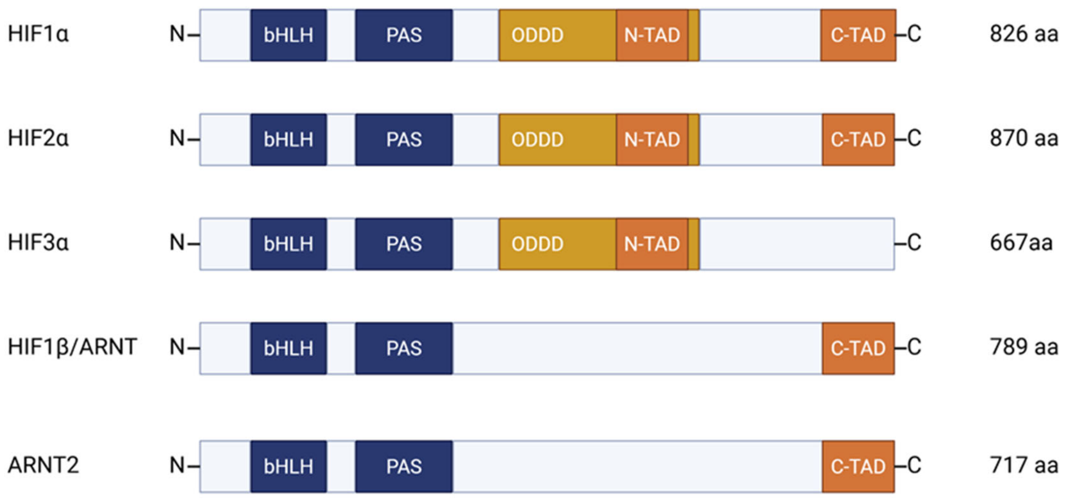

Figure S1. Schematic representation showing the domain arrangement of HIFs. Hypoxia inducible factor subunits contain several common domains, including basic helix-loop-helix (bHLH) domains, Per-Arnt-Sim (PAS) domains, N-terminal transactivation domain (N-TAD), a C-terminal transactivation domain (C-TAD), and an oxygen-dependent domain (ODDD). This figure depicts the 3 paralogs of the HIF-α subunit (HIF-1α, HIF-2α, HIF-3α) and the 2 paralogs of the HIF-β subunit (HIF-1β/ARNT and ARNT-2). This representative image does not depict all major domains of HIFs or represent the multiple isoforms of HIFs. Created in BioRender. Dingley, R. (2026) https://BioRender.com/0gbyqy0.

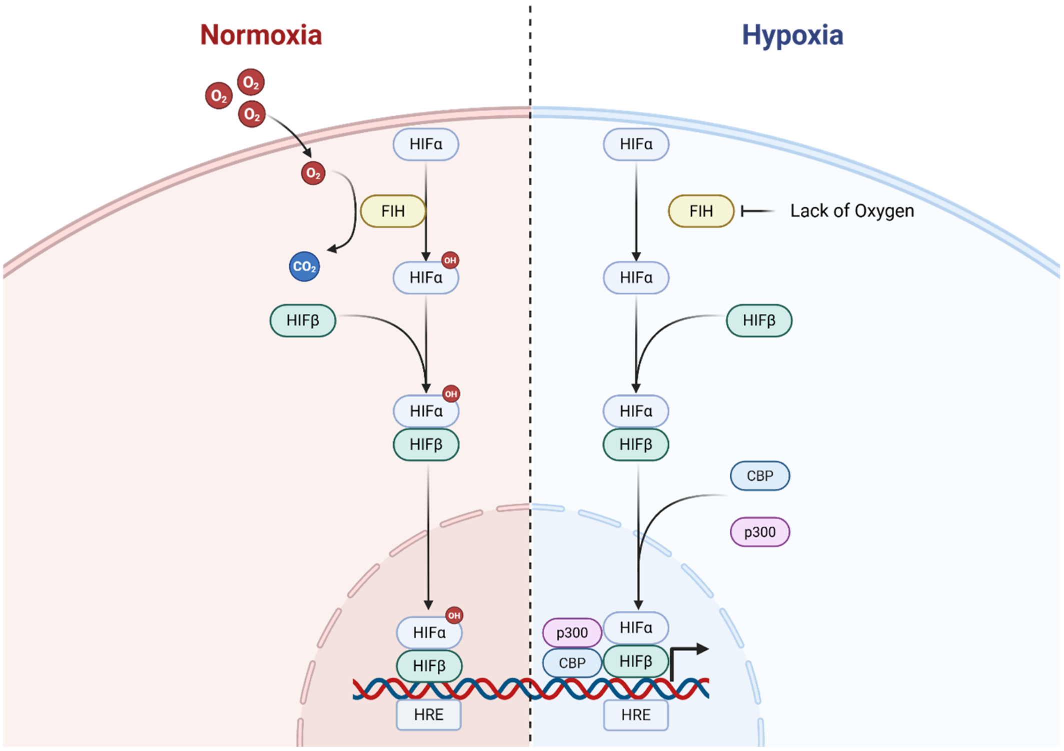

Figure S2. Schematic of the HIF oxygen-dependent inhibition pathway. Under normoxic conditions, the asparagine residues of HIF-α subunits are hydroxylated by Factor-Inhibiting Hypoxia-Inducible Factor (FIH), inhibiting the binding of key cofactors required for transcriptional activation. Under hypoxic conditions, the low availability of O_2 inhibits FIH activity, thereby allowing co-factor binding and transcriptional activation of target genes containing hypoxia response elements (HREs). Created in BioRender. Dingley, R. (2026) https://BioRender.com/6t52y3t.
